# Reduced *PRF1* enhancer methylation in children with a history of severe RSV bronchiolitis in infancy: an association study

**DOI:** 10.1186/s12887-017-0817-9

**Published:** 2017-03-03

**Authors:** Magdeldin Elgizouli, Chad Logan, Ruth Grychtol, Dietrich Rothenbacher, Alexandra Nieters, Andrea Heinzmann

**Affiliations:** 1 Center for Chronic Immunodeficiency, Medical Center – University of Freiburg, Faculty of Medicine, University of Freiburg, Breisacherstr. 115 4, Freiburg, D-79106 Germany; 2 Institute of Epidemiology and Medical Biometry, Ulm University, Ulm, Germany; 3 Department of Pediatrics and Adolescent Medicine, Medical Center – University of Freiburg, Faculty of Medicine, University of Freiburg, Freiburg, Germany

**Keywords:** Respiratory syncytial virus, Infant bronchiolitis, DNA methylation, Perforin, *PRF1*

## Abstract

**Background:**

Acute lower respiratory tract infection is the commonest disease affecting children under five worldwide. Respiratory syncytial virus (RSV) is among the most common causative pathogens. Epidemiological data suggest an association between severe viral respiratory infections in infancy and increased incidence of childhood wheeze and asthma. DNA methylation is involved in immune cell differentiation and identity. It provides an avenue for environmental influences on the genome and therefore has potential as a marker for sustained effects of infectious insults. In this study we investigated the association between DNA methylation patterns in the perforin gene (*PRF1*) in childhood and a history of hospitalisation for severe RSV disease in the first two years of life.

**Methods:**

In this retrospective study, we explored patterns of whole blood DNA methylation at a methylation sensitive region of the proximal *PRF1* enhancer in a group of children with a record of hospitalisation for severe RSV disease during infancy (*n* = 43) compared to healthy controls matched for age and sex with no similar hospitalisation history, no allergy and no persistent wheeze (*n* = 43). Univariate and bivariate conditional logistic regression analyses were conducted to test the association between *PRF1* enhancer methylation and record of hospitalisation for RSV disease.

**Results:**

Children with a record of hospitalisation for severe RSV bronchiolitis demonstrated markedly lower levels of DNA methylation at two cytosine-phosphate-guanine dinucleotide (CpG) loci of the *PRF1* proximal enhancer, corresponding to a signal transducer and activator of transcription 5 (STAT5) responsive element, compared to controls, adjusted odds ratios of 0.82 (95% confidence interval [CI] 0.71, 0.94) and 0.73 (95% CI 0.58, 0.92) for each 1% increase in DNA methylation. Smoking in the household showed a significant influence on DNA methylation at the assayed positions.

**Conclusions:**

Our findings support an association between childhood DNA methylation patterns in *PRF1* and a record of severe RSV infection in infancy. Longitudinal studies are required to establish the utility of *PRF1* methylation as a marker of severe RSV disease.

## Background

Acute lower respiratory tract infection (LRTI) is the leading cause of child mortality worldwide, the greater burden falling on developing countries [1]. On a global scale, respiratory syncytial virus (RSV) is the most common causative pathogen of LRTI in children under five years old and a major cause of hospital admission particularly in the first year of life. The incidence in infants is three times higher than overall incidence in children under five years of age [2].

Evidence is strong that viral respiratory infections in infancy are associated with increased risk of childhood wheezing, atopy and asthma [3], up to adolescence [4] and possibly early adulthood [5]. It is not entirely clear whether viral LRTIs including RSV are indeed a causal factor or simply indicate heightened susceptibility to asthma although considerable evidence supports a causal link [6].

DNA methylation, the commonest epigenetic alteration known to date, is crucial to processes of cellular differentiation and the establishment of cellular identity [7]. In contrast to genetic hardwire, genome-wide patterns of DNA methylation and other chromatin modifications do not persist throughout life but undergo specific changes at defined stages of development that contribute to lineage and tissue-specific gene expression [8]. As such, epigenetic modifications constitute a dynamic interface between genome and environment. Epigenetic modifications have been characterized in key effector and regulator genes as determinants of cell type and function in the immune system and modulators of immune response [9].

Epigenetic dysregulation is one potential mechanism which may mediate between adverse in utero and early life exposures such as severe infections and immunological functions in later life [10]. The interactions mediating the relationship between severe RSV bronchiolitis and adverse consequences in childhood are broadly understood to be immunological in nature [11] but are yet to be fully uncovered. Perforin is a potent cytolytic molecule of cytotoxic T cells and natural killer (NK) cells capable of non-specific lysis of virus-infected cells [12]. Perforin expression is under the control of interleukin (IL)-2, signalling through signal transducer and activator of transcription (STAT)-5 [13].

We previously reported higher methylation of specific cytosine-phosphate-guanine dinucleotide (CpG) positions corresponding to a STAT5 binding element in an enhancer region of *PRF1* in cord blood DNA of infants who subsequently developed frequent LRTI during their first year of life [14]. Considering the role perforin plays in the cellular immune response to virus infections, we hypothesised that severe RSV infection in infancy might be associated with alterations in *PRF1* methylation. Accordingly, we assayed DNA methylation at the two CpGs of the STAT5 element in the *PRF1* enhancer, in a cohort of children with a history of severe RSV bronchiolitis requiring hospitalisation during infancy compared to healthy children matched by sex and age at blood collection with no record of severe bronchiolitis, allergic disease or persistent wheeze.

## Methods

### Study subjects

*Children with history of severe RSV disease* were drawn from a cohort of children recruited at the University Children’s Hospital and St Josef’s Community Children Hospital in Freiburg, Germany, between September 1998 and March 2005 for the primary purpose of investigating genetic associations with severe RSV disease [15].

All children who were hospitalised for severe RSV disease in the first two years of life were considered eligible for inclusion in the cohort. Cases had symptoms of bronchiolitis at admission, such as wheezing and tachypnoea, and needed either supplementary oxygen and/or gavage feeding and/or intravenous fluids. Infection with RSV was confirmed by antigen test and/or polymerase chain reaction (PCR). Parents of all eligible patients were approached to enrol in the study and included upon informed consent.

Children with congenital heart defects, immunodeficiency, or chromosomal aberrations were excluded from the study. Information on treatment course was obtained from patients’ hospital records regarding requirement for corticosteroid, antibiotic and oxygen therapy as well as a record of persistent wheeze. Indicators of family environment such as number of siblings and parents smoking in the house were obtained via standardized questionnaire filled in by the parents at time of blood draw. Blood samples were collected from study participants at a median of 3.25 years after hospitalisation (IQR 3.16–4.00). From a total baseline study population of 208 cases blood samples were obtained from 131 cases.

*Healthy controls matched to cases by sex and approximate ages* were recruited from healthy children seen at several paediatric practices in the same catchment area of Freiburg in southwestern Germany. Controls were recruited in the same time period as cases, 1998 to 2005, for the original purpose of the genetic association studies mentioned. Controls had no record of severe bronchiolitis in the first two years of life and were never diagnosed with persistent wheeze or allergic disease. Sex and age matching was not considered at time of recruitment but was undertaken for the purpose of this study. Controls were matched 1:1 on sex and the same age at blood sampling (plus or minus one year).

Blood samples were collected in ethylenediaminetetraacetic acid (EDTA) tubes processed, aliquoted and stored at −20 °C. Methylation assays were performed on genomic DNA derived from peripheral blood leukocyte samples of 43 children with a history of severe RSV disease and 43 healthy children with no history of severe RSV disease.

Collection of samples and study protocols were approved by the Ethics Commission of the Medical Centre, University of Freiburg. Written informed consent was obtained from parents of all participating children.

### DNA extraction and bisulfite modification

Genomic DNA was manually extracted from peripheral blood leukocytes using the QIAamp® DNA Blood Midi Kit (Qiagen GmbH, Hilden, Germany) according to the manufacturer’s protocol. DNA was stored at −20 °C before use. In preparation for pyrosequencing, 500 ng of DNA was bisulfite converted using the EZ DNA Methylation-Gold™ Kit (Zymo Research Corp, Irvine, CA, USA) according to the manufacturer’s protocol. Non-methylated cytosines in this procedure are converted to uracil which is replaced in subsequent amplification by thymine while methylated cytosines are preserved unchanged.

### Target cytosine dinucleotides

**Fig. 1:**
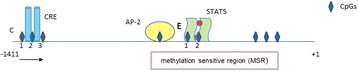
The proximal *PRF1* enhancer and binding sites for transcription factors. Individual CpGs marked by diamond shapes. Assayed CpGs identified as E 1 and 2. CRE: cAMP responsive element; AP2: activating protein 2; STAT5: signal transducer and activator of transcription [14]

Cytosine methylation levels were measured in two CpG dinucleotides in the methylation sensitive region (MSR) of *PRF1* located 929 to 577 base pairs (bp) upstream of the transcription start site (TSS) and referred to as CpGs E1 and E2 as detailed previously [14]. Figure 1 shows the two CpGs investigated in the MSR of the *PRF1* proximal enhancer.

### Bisulfite pyrosequencing

Quantification of cytosine percent methylation was performed by pyrosequencing of bilsulfite-converted DNA. Primers were designed with the PyroMark Assay Design Software v.2.0.1.15 (Qiagen GmbH, Hilden, Germany) to amplify the target region and purchased from Apara Biosciences GmbH (Denzlingen, Germany). Each assay included non-CpG cytosines as internal controls to verify efficient bisulfite conversion.

1.5 pmol each of the forward primer 5′-GAGTGGGAGAAGAGAGATG-3′ and the reverse biotinylated primer 5′-CCCCCAATACCCTATAAATCACTC-3′ were used to amplify the fragment of interest, sized 256 base pairs (bp), from an input amount of 0.5 μl bisulfite-converted DNA, 25 ng assuming complete recovery from bisulfite conversion, in a final reaction volume of 12.5 μl using the PyroMark PCR Kit (Qiagen GmbH, Hilden, Germany). PCR conditions were as follows 95 °C for 10 min; 50 cycles of 94 °C for 30 s, annealing for 30s, 72 °C for 30 s; and a final extension step at 72 °C for 10 min.

PCR products were rendered single stranded according to established protocol [16]. 3 pmol of the sequencing primer 5′-GTGAGGATAGTTAGTGG-3′ were used to perform the pyrosequencing reaction on the PyroMark Q96 MD apparatus (Qiagen GmbH, Hilden, Germany). The percentage methylation at each CpG was calculated using the PyroMark CpG Software v.1.0.11 build 14 (Qiagen GmbH, Hilden, Germany). The quantitative performance of the pyrosequencing assay was assessed by measuring methylation standards of known proportions of unmethylated (whole genome amplified) and fully methylated (*Sss-I* treated) genomic DNA and optimized by means of an annealing temperature gradient. Matched cases and controls were consistently assayed in the same batch. All samples were assayed in duplicates and the mean percentage of the two measurements calculated and used for statistical analyses.

### Statistical analysis

Univariate analyses were conducted on each candidate gene locus to assess methylation results for normality and identify potential outliers. Methylation measurements greater than 3 standard deviations from the mean were considered as potential outliers. To identify potential confounding factors not accounted for by matching, age at sampling, gestational age (<37 weeks or ≥37 weeks), household smoking (yes or no), and whether or not the child had a sibling under 5 years of age (yes or no) were assessed for association with RSV case status and methylation levels using non-parametric testing methods (Chi-square, Fisher’s exact, Spearman’s coefficients and Kruskall-Wallis tests). Selection of potential confounders was based on a *p*-value ≤ 0.05. Household smoking was associated with both RSV case status and methylation levels and thus considered a potential confounding factor for adjustment in logistic regression models. Bivariate and adjusted conditional logistic regression models were used to calculate the odds ratio (OR) per 1% difference in methylation, 95% confidence interval (CI), and p-value for the association with case status at each candidate gene locus after adjustment for age at blood sampling and household smoking. In order to determine the effect of potential outliers on the results, a second bivariate analysis was performed restricted to pairs (case and matched control) containing no outlier values. All statistical analyses were performed using SAS 9.3 for Windows (SAS Institute, Cary, US).

## Results

### Study population

*RSV cases and controls* (*n* = 86) were 1:1 matched according to sex and approximate age at time of blood draw. Features of the RSV case-control population are presented in Table 1. Overall, children were predominantly male (65%). RSV cases had a median age of 3.3 years (interquartile range [IQR] 3.2, 4) while controls had a median age of 4 years (IQR 4.0, 5.0). The majority of children were born term, 81.1% of cases and 87.5% of controls. Cases had more often siblings and were more exposed to household smoking compared to controls (Table 1).

**Table 1:** Characteristics of children with history of hospitalisation for RSV bronchiolitis and healthy controls

	RSV-cases		Controls		*p*-value*
	n	% or median (p25, p75)	n	% or median (p25, p75)	
Age at blood sampling (years)	43	3.3 (3.2, 4.0)	43	4.0 (4.0, 5.0)	<.001
Gender					1
Female	15	34.9%	15	34.9%	
Male	28	65.1%	28	65.1%	
Gestational age					0.53
≥37 weeks	30	81.1%	28	87.5%	
<37 weeks	7	18.9%	4	12.5%	
Sibling status					0.02
None	2	5.7%	9	29%	
≥1	33	94.3%	22	71%	
Smoking in household					0.02
No	19	47.5%	24	75%	
Yes	21	52.5%	8	25%	

*p-value calculated as either chi-square, Fisher’s exact, or Kruskal-Wallis test

Among the cases 70% had a complicating bacterial infection requiring antibiotic medication, 30% received corticosteroid therapy and 10% already had a persistent wheeze before hospitalisation for RSV infection. Hospitalisation for severe RSV disease was associated with exposure to smoking in the household (*p* = 0.02) and having one or more siblings (*p* = 0.02).

### Differential DNA methylation at the *PRF1* enhancer

Among all subjects, exposure to smoking in the household was associated with lower DNA methylation levels at E1 and E2 (*p* = 0.003, 0.004) but no associations were observed for gestational age, sex, or sibling status. Among RSV cases only (for whom more robust data was available), a diagnosis of persistent wheeze prior to hospitalisation for RSV disease was associated with lower DNA methylation levels in E1 (*p* = 0.04). None of the other clinical characteristics explored showed a statistically significant association with DNA methylation at the *PRF1* loci except maternal asthma (*p* = 0.04 with E2) (Table 2). When cases and controls were combined, exposure to smoking in the household was associated with lower DNA methylation levels at E1 and E2 (*p* = 0.003, 0.004) but no associations for gestational age, sex, and sibling status remained non-significant (Table 3).

**Table 2:** Differential *PRF1* enhancer methylation according to clinical features of RSV bronchiolitis among cases only

Characteristics	DNA methylation levels at the *PRF1* enhancer					
	CpG E1			CpG E2		
	n	median (p25, p75) or Spearman’s coefficient	*p*-value*	n	median (p25, p75) or Spearman’s coefficient	*p*-value*
Age at hospitalisation (years)	38	−014	0.42	38	−016	0.33
Age at blood sampling	43	0.08	0.61	43	0.10	0.54
Gender			0.79			0.30
Female	15	68.0 (62.5, 73.5)		15	67.0 (63.0, 70.0)	
Male	28	69.5 (61.0, 74.5)		28	64.8 (59.3, 70.0)	
Gestational age			0.32			0.98
≥37 weeks	30	68.3 (63.0, 73.5)		30	64.8 (62.0, 68.5)	
<37 weeks	7	62.0 (36.0, 73.5)		7	65.5 (36.0, 72.0)	
Sibling status			0.62			0.57
None	2	55.3 (36.0, 74.5)		2	53.0 36.0, 70.0)	
≥1	33	69.5 (63.0, 74.5)		33	65.5 (63.0, 71.5)	
Smoking in the household			0.08			0.14
No	19	72.0 (66.0, 74.0)		19	67.0 (63.5, 72.0)	
Yes	21	65.5 (61.0, 70.0)		21	64.0 (61.5, 68.5)	
Previous wheeze			0.04			0.13
No	36	69.5 (63.8, 74.5)		36	65.5 (62.5, 71.0)	
Yes	4	62.3 (43.5, 64.3)		4	63.3 (42.0, 64.5)	
Parent allergic disease			0.50			0.23
No	20	69.5 (63.0, 75.0)		20	67.0 (63.5, 71.8)	
Yes	20	68.8 (62.3, 72.8)		20	64.0 (61.8, 69.3)	
Paternal asthma			0.07			0.19
No	37	69.5 (63.0, 74.5)		37	65.0 (63.0, 70.5)	
Yes	3	62.0 (25.0, 66.0)		3	63.5 (21.0, 65.5)	
Maternal asthma			0.32			0.04
No	37	69.5 (63.0, 74.5)		37	65.5 (63.0, 70.5)	
Yes	3	66.0 (25.0, 70.0)		3	61.5 (21.0, 63.5)	
Days of oxygen supplementation	40	−0.05	0.74	40	−0.11	0.51
Antibiotic therapy			0.62			0.65
No	12	69.5 (46.8, 74.5)		12	65.0 (46.5, 71.8)	
Yes	28	69.5 (63.8, 74.0)		28	65.0 (63.5, 69.5)	
Corticosteroid therapy			0.38			0.32
No	28	67.8 (61.8, 74.0)		28	64.8 (61.5, 70.3)	
Yes	12	71.3 (65.0, 74.0)		12	66.5 (64.0, 71.0)	

Differences in the total number of observations are due to missing data

*n* number of observations, *p25* 25th percentile, *p75* 75th percentile

*p-value for categorical variables calculated using Kruskal-Wallis test

**Table 3:** Differential *PRF1* enhancer methylation according to characteristics of RSV bronchiolitis among all subjects (cases and controls combined)

Characteristics	DNA methylation levels at the *PRF1* enhancer					
	CpG E1			CpG E2		
	n	median (p25, p75)	*p*-value*	n	median (p25, p75)	*p*-value*
Gender			0.06			0.09
Female	30	74.8 (66.0, 83.0)		30	73.3 (67.0, 78.5)	
Male	56	74.0 (60.0, 78.8)		56	70.8 (64.3, 74.5)	
Gestational age			0.26			0.61
≥37 weeks	58	74.5 (67.5, 80.0)		58	70.8 (64.0, 75.0)	
<37 weeks	11	73.5 (55, 75.5)		11	70.0 (55.0, 73.5)	
Sibling status			0.34			0.25
None	11	75.5 (74.5, 84.5)		11	73.0 (70.0, 78.5)	
≥1	55	74.5 (66.0, 80.0)		55	71.5 (64.0, 75.0)	
Smoking in the household			0.003			0.004
No	43	75.5 (70.0, 83.2)		43	72.0 (67.0, 78.5)	
Yes	29	69.5 (62.5, 76.0)		29	68.0 (63.0, 72.5)	

Differences in the total number of observations are due to missing data

*n* number of observations, *p25* 25^th^ percentile, *p75* 75^th^ percentile

*p-value calculated using Kruskal-Wallis test

Median DNA methylation levels of 69.5% (IQR 62.5, 74.5) and 65.0% (IQR 62.0, 70.0) were observed at CpGs E1 and E2 (illustrated in Fig. 1) in children hospitalised for RSV disease compared to 79.0% (IQR 75.0, 84.0) and 75.0% (IQR 72.0, 79.0) in controls (*p* < 0.001). Logistic regression analysis adjusted for exposure to smoking in the household demonstrated OR of 0.82 (95% CI 0.71, 0.94) and 0.73 (95% CI 0.58, 0.92) for E1 and E2 respectively for each 1% increase in methylation in association with case status (Table 4).

**Table 4:** Crude and adjusted model results for association between *PRF1* enhancer methylation and previous RSV bronchiolitis

*PRF1* CpG position	Model			
	Crude		Adjusted^a^	
	Odds Ratio (95% CI)^†^	*p*-value	Odds Ratio (95% CI)^†^	*p*-value
E1	0.81 (0.71, 0.92)	<0.001	0.82 (0.71, 0.94)	0.010
E2	0.67 (0.52, 0.87)	0.003	0.73 (0.58, 0.92)	0.009

^a^adjusted for household smoking

^†^Odds ratio for risk of case status based on a 1% increase in PRF1 methylation level and case status

In an additional sensitivity analysis, adjustment for age at blood draw was performed to account for the loose age matching between cases and controls. Adjustment for age at blood sampling alone did not alter the point estimates. The small sample size however did not allow adjustment for both household smoking exposure and age at blood sampling.

## Discussion

We investigated the whole blood DNA methylation pattern of two CpG sites in the proximal enhancer of the immune effector gene *PRF1* in a group of children with a history of hospitalisation for RSV disease in infancy compared to healthy children of matched sex and approximate age at blood sampling. In cases with RSV history we found markedly lower levels of DNA methylation at two loci of the *PRF1* proximal enhancer. In addition, infants who were admitted for RSV disease were predominantly male, had one sibling or more, and were more likely to come from a household where an adult smoked.

Holberg and colleagues reported a significant association of male sex with severe RSV infection in the first three months of life in the Tucson Children’s Respiratory Study [17], an association confirmed in multiple other studies [18]. Having a sibling has been repeatedly reported as a risk factor for RSV induced LRTI in infancy [19]. The association with passive exposure to cigarette smoke is less consistent [18] but appears strong in combination with prematurity [20]. Infants exposed to postnatal maternal smoking suffer a more severe course of RSV disease with lower oxygen saturation during hospitalisation than the non-exposed [21]. In a large-scale surveillance of US children under five years spanning four years Hall and co-workers identified only age under six months and history of prematurity as independent risk factors for RSV hospitalisation [22]. The discrepancy may be related to the wider age definition in the latter study.

From the variables available for this analysis only exposure to smoking in the household showed an association with DNA methylation at the *PRF1* enhancer. Smoking is a potent environmental modulator of genome-wide DNA methylation including in utero, when methylation patterns are analysed in cord blood [23]. The association we observed between reduced DNA methylation at the *PRF1* enhancer in whole blood three to four years after hospitalisation for severe RSV disease during infancy remained significant after adjustment for smoking exposure. A separate analysis adjusted for age was performed to address the loose age matching in the study population but showed no significant difference in estimates.

The two CpGs assayed are located in a segment of the *PRF1* enhancer characterised as a methylation sensitive segment. Methylation of the *PRF1* MSR results in suppression of *PRF1* while demethylation is associated with heightened expression in T cells [24]. Our analysis was performed on DNA derived from whole blood, and we cannot with the available measurements rule out a confounding effect of cell subtype composition on our measurements. Hence, our results might reflect an expansion of the perforin-positive lymphocyte population rather than a true demethylation event in otherwise *PRF1*-methylated cells or more likely a combination of both. Perforin is normally expressed in almost all NK cells, NKT cells, γα T cells and around 20–30% of cytotoxic T cells and is broadly considered a marker of effector CD8+ T cell activation [25].

CD8+ T cell proliferation and differentiation from naïve to effector and memory T cells in response to virus infection is associated with distinct epigenetic profiles corresponding to cell-specific transcriptional programmes [26]. We previously reported a prospective association between increased methylation at the *PRF1* enhancer MSR in cord blood and LRTI in the first year of life suggesting a reduced T cell/perforin reaction [14]. A robust cell-mediated cytotoxic response is necessary for viral clearance of RSV infection [27] but failure to effectively regulate this reaction might contribute to T cell mediated lung injury [28].

Accordingly, the reduced DNA methylation we observe in this retrospective study in contrast to the increased DNA methylation at the same locus in our prospective study of cord blood *PRF1* methylation and LRTI risk in the first year of life might reflect the differing requirements for a cytotoxic T cell response in these two situations. A robust cytotoxic response is needed to protect against respiratory viral infection, and reduced *PRF1* expression in newborns as indicated by higher DNA methylation is associated with increased risk of LRTI. On the other hand, increased expression of *PRF1* as indicated by reduction in DNA methylation, is associated with a record of severe RSV infection, and might contribute to lung damage. For instance, Arnold et al. reported an increase in perforin-positive peripheral blood lymphocytes in both intrinsic and allergic asthma [29].

Within the group of children with a history of RSV hospitalisation, we observed a greater reduction of whole blood DNA methylation at the *PRF1* enhancer in a subgroup who had a persistent wheeze before hospitalisation. Accordingly, we assayed the same locus in a separate group of asthmatic children and could observe intermediate levels of DNA methylation when compared to the higher levels in controls and lower levels in children with a history of hospitalisation for RSV disease (data not shown). Our analysis of asthmatic children (6 years old) is hampered by the lack of exact age-matched healthy controls which may have resulted in spurious results.

The cross-sectional design of this study does not allow for conclusions regarding temporal changes in DNA methylation. Only a longitudinal analysis would establish whether the reduced DNA methylation levels we observed are restored as the affected children grow. Therefore, further research may be warranted to establish causality. Childhood is a period of intense age-related changes in peripheral blood DNA methylation with a three to fourfold higher rate of change compared to adulthood [30].

Recurrent wheezing is a common complication of severe RSV bronchiolitis in infancy Meta-analysis of longitudinal studies however suggests that the heightened risk of wheeze and/or asthma is abolished by the fifth year of follow up [31]. DNA methylation is discussed as a mechanism of gene-environment interplay in the pathogenesis of asthma [32]. Several candidate gene and genome-wide association studies have so far documented differential DNA methylation patterns in asthma patients, including in T cells [33] and airway epithelial cells [34].

When looking at the results, the following limitations should be considered: Due to difficulty in obtaining a larger number of sex and age matched controls our study consisted of only 86 subjects. We had only limited additional data on our subjects to test for potential confounding. Though we were able to rule out confounding by two important factors previously associated with severe RSV infection (presence of smoking in the household and additional siblings in the household), the limited sample size did not allow for a thorough investigation of potential confounders. We could not completely rule out the possibility that the association we observed was due to chance selection of discordant subjects. Finally, due to the retrospective nature of the case-control design and measurement of only blood DNA methylation at one point in time, it remains unclear whether methylation levels were affected already prior to RSV infection. To further explore our preliminary findings a longitudinal analysis of a larger cohort with the possibility of isolating lymphocyte subtypes backed by sufficient epidemiological exposure data would be required.

## Conclusions

We observed a reduction in blood DNA methylation of the *PRF1* proximal enhancer in children in association with previous hospitalisation for severe RSV bronchiolitis during infancy. This pattern of differential DNA methylation is worthy of investigation as a candidate marker for complications of severe RSV infection particularly persistent wheeze and childhood asthma. Our findings underscore the importance of considering the temporal dimension when studying DNA methylation patterns. Furthermore, they highlight the need for well-matched adequately powered longitudinal studies of epigenome-wide DNA methylation which, when supplemented with further epidemiological data, may improve our understanding of the dynamics of gene-environment interaction in the pathogenesis of disease.

## Abbreviations

Bp: Base pair
CI: Confidence interval
CpG: Cytosine-phosphate-guanine
IQR: Interquartile range
LRTI: Lower respiratory tract infection
MSR: Methylation sensitive region
NK: Natural killer
OR: Odds ratio
PRF: Perforin
RSV: Respiratory syncytial virus
STAT5: Signal transducer and activator of transcription 5
TSS: Transcription start site
